# CX3CR1 identifies a potent effector CD8^+^ T cell subset associated with anti-PD-1 therapeutic efficacy in colorectal cancer

**DOI:** 10.3389/fimmu.2026.1770119

**Published:** 2026-03-18

**Authors:** Jiajin Ma, Yue Wu, Rongzhang Chen, Nuo Wang, Yirui Liu, Lujun Chen, Shaoxian Wu, Xiao Zheng

**Affiliations:** 1 Department of Tumor Biological Treatment, The Third Affiliated Hospital of Soochow University, Changzhou, Jiangsu, China; 2 Jiangsu Engineering Research Center for Tumor Immunotherapy, The Third Affiliated Hospital of Soochow University, Changzhou, Jiangsu, China; 3 Institute of Cell Therapy, The Third Affiliated Hospital of Soochow University, Changzhou, Jiangsu, China

**Keywords:** colorectal cancer, CX3CR1+CD8+T cells, immunotherapy, multi-color immunohistochemical staining, prognosis

## Abstract

**Background:**

The therapeutic landscape for deficient mismatch repair (dMMR) colorectal cancer (CRC) has been fundamentally transformed by the introduction of immune checkpoint inhibitors (ICIs). Yet, a significant fraction of patients exhibits primary or acquired resistance, underscoring a critical unmet need to decode the heterogeneity of tumor-infiltrating CD8^+^T cells. Identifying the effector subsets that truly drive tumor control will be essential for refining patient stratification and optimizing therapeutic interventions.

**Methods:**

By leveraging a multi-dimensional approach that combined single-cell RNA sequencing (scRNA-seq), multiplex immunohistochemistry (mIHC), extensive clinical data mining, and *in vivo* murine models, we comprehensively delineated the phenotypic profile, regulatory networks, and clinical relevance of intratumoral CX3CR1^+^CD8^+^T cells.

**Results:**

We uncovered a unique CX3CR1^+^CD8^+^ T cell subset exhibiting a Temra-like terminal effector phenotype characterized by potent cytotoxicity (GNLY, PRF1) and low exhaustion. Clinically, enrichment of this population emerged as a strong, independent predictor of improved overall survival in CRC. Mechanistically, effective anti-PD-1 therapy revitalizes anti-tumor immunity by specifically promoting the proliferation and maintaining the function of these CX3CR1^+^ effector cells within the tumor. SCENIC analysis further revealed that this differentiation pathway is orchestrated by *ETS1*, *PRDM1* and *STAT1* driven regulatory network, which is markedly induced following PD-1 inhibition to enhance metabolic fitness and cellular recruitment.

**Conclusion:**

This study characterizes CX3CR1^+^CD8^+^T cells as a key effector subset predicting prognosis and immunotherapy response in colorectal cancer. These insights not only support their use as a clinically meaningful biomarker but also highlight the regulatory network orchestrated by *ETS1* and *PRDM1* as a promising avenue for overcoming immune resistance.

## Introduction

CRC remains a leading cause of cancer mortality, projected to rank third in incidence in 2025 (1). Despite advances in surgical and systemic therapies, prognosis for advanced disease remains suboptimal, with 5-year survival rates of only 40% for oligometastatic and approximately 20% for metastatic cases (2–7). Consequently, immune checkpoint blockade, specifically targeting the PD-1/PD-L1 pathway, has established itself as a cornerstone of modern oncology (8, 9). While the approval of immune checkpoint inhibitors (ICIs) for microsatellite instability-high (MSI-H)/dMMR tumors marked a paradigm shift in precision oncology (10–12), clinical benefits are restricted. The MSI-H phenotype comprises only about 15% of CRC cases, dropping to 4–5% in metastatic disease (13, 14). Conversely, the predominant microsatellite stable (MSS) phenotype typically manifests as immunologically ‘cold’ and refractory to ICIs (15). Moreover, therapeutic efficacy is not guaranteed even within the MSI-H cohort, where approximately 30% to 50% of patients manifest either primary refractoriness or acquire resistance to single-agent anti-PD-1 treatment (11, 16). Thus, dissecting the heterogeneity of the tumor microenvironment (TME) to identify specific T cell subsets driving antitumor immunity is imperative for overcoming resistance and refining patient stratification.

The clinical efficacy of PD-1 inhibition hinges on the revitalization of CD8^+^ cytotoxic T lymphocytes (CTLs) and the reversal of microenvironmental immunosuppression. Mechanistically, PD-1 blockade not only relieves direct inhibitory signaling on TCR and CD28 but also drives metabolic reprogramming, enabling T cells to regain mitochondrial fitness and persist within the nutrient-deprived TME (17). Furthermore, this revitalization drives a reciprocal interaction with the TME, whereby reinvigorated T cells release pro-inflammatory cytokines such as IFN-γ. This secretion subsequently prompts stromal and myeloid cells to produce chemokines like CXCL9 and CXCL10, establishing a self-reinforcing feedback loop that amplifies the infiltration of specific effector T cell subsets into the tumor bed (18, 19). Indeed, the density of these effectors, quantified as the Immunoscore, offers superior prognostic accuracy compared to traditional TNM staging (20–22). However, tumor-infiltrating lymphocytes (TILs) exhibit profound heterogeneity. Chronic antigen exposure typically drives T cells into a dysfunctional exhausted state, characterized by the upregulation of inhibitory molecules (e.g., PD-1, TIM-3) and impaired cytotoxicity (23, 24). While revitalizing exhausted T cells is the primary target of ICIs, recent high-dimensional single-cell analyses suggest that therapeutic benefit may also depend on the recruitment and maintenance of specific non-exhaustion effector subsets (25, 26). Consequently, distinguishing bona fide effectors from terminally exhausted populations is critical for elucidating the mechanisms of ICIs responsiveness.

CX3CR1 acts as a canonical G protein-coupled receptor (GPCR), mediating signal transduction through its interaction with heterotrimeric G proteins. Structurally, this 355-amino acid protein is characterized by a seven-transmembrane α-helical architecture. Its topology includes an extracellular N-terminus and loops responsible for binding the ligand CX3CL1, alongside an intracellular C-terminus and loops that form the docking site for G protein α subunits (27, 28). Genetically, the *CX3CR1* locus maps to chromosome 3p22.2, comprising four exons and three introns, with transcriptional activity tightly governed by three distinct upstream promoters: P1, P2, and P3 (29). Surpassing its fundamental molecular characteristics, CX3CR1 is now widely acknowledged as a robust indicator of T cell differentiation status (30). Within the settings of chronic viral infection and malignancy, the expression of CX3CR1 identifies a specific subpopulation of effector memory T cells (Tem). These cells are distinguished from both stem-like progenitors and terminally exhausted populations by their potent cytolytic capacity despite a limited potential for proliferation (31, 32). Emerging evidence from clinical cohorts in melanoma and non-small cell lung cancer (NSCLC) suggests that the frequency of CX3CR1^+^CD8^+^T cells, whether circulating or tumor-infiltrating, correlates positively with therapeutic efficacy following PD-1 inhibition (33, 34). Recently, comprehensive pan-cancer single-cell atlases have broadly characterized CX3CR1^+^CD8^+^ T cells as a terminal effector subset distinct from exhausted populations (35, 36). However, the specific transcriptional regulatory networks governing the generation and maintenance of this subset within the unique immune microenvironment of CRC, and its precise clinical value in stratifying heterogeneous populations (e.g., MSI-H vs. MSS), remain to be fully elucidated.

In this study, we employed an integrated multi-omics approach by combining scRNA-seq, mIHC, and large-scale clinical cohort profiling to systematically characterize the landscape of CX3CR1^+^CD8^+^T cells within the CRC tumor microenvironment. We identified this subset as a distinct population of non-exhausted, terminally differentiated effector memory (Temra) cells, distinguished by potent cytotoxicity and minimal exhaustion features. Clinically, robust intratumoral infiltration of this subset served as an independent prognostic factor associated with prolonged overall survival (OS). Mechanistically, utilizing *in vivo* models and SCENIC inference, we demonstrated that PD-1 blockade reinvigorates antitumor immunity by driving the intratumoral expansion and functional persistence of these effectors via a transcriptional regulatory network orchestrated by *ETS1* and *PRDM1*. Collectively, these findings highlight the CX3CR1^+^ subset as a pivotal determinant of immunotherapy efficacy, providing a rationale for targeting this population to overcome resistance in CRC.

## Materials and methods

### Patient cohort and tissue samples

A high-density CRC tissue microarray (TMA) was obtained from Shanghai Outdo Biotech Co., Ltd. (Shanghai, China) and employed for the present investigation. The specific product identifiers are Cat. No. HColA180Su22 and Protocol No. XCSW-JS1-SOP-002-F0. The original cohort comprised 94 pathologically confirmed CRC tumor tissue cores and 86 matched adjacent non-tumor tissue cores. In order to guarantee the robustness of our prognostic evaluation, we implemented rigorous eligibility standards. The cohort was comprised exclusively of treatment-naive individuals, meaning no patients had undergone preoperative neoadjuvant chemotherapy, radiotherapy, or any other forms of anti-tumor interventions. Comprehensive clinicopathological parameters were retrieved for each patient, including gender, age, tumor dimensions, differentiation grade, and TNM staging. The cohort covered surgical resections performed between February 2012 and November 2014, with clinical follow-up extending through June 2021. The final study cohort consisted of 78 patients, after those with missing clinical information, loss to follow-up, or damaged tissue cores were removed from the dataset. Approved by the Clinical Research Ethics Committee of Shanghai Outdo Biotech Co., Ltd. (No. SHYJS-CP-230902), all research procedures were performed in strict accordance with relevant ethical standards.

### Multi-color immunohistochemically staining

The spatial co-expression of PANCK, CD8, and CX3CR1 in CRC tissues was evaluated via multi-color immunohistochemically (mIHC) staining (AlphaX TSA 7-Color Kit, Cat. No. AXT37100041) per standard protocols. The slides were first heated to 63 °C for 60 min, followed by automated dewaxing and rehydration using xylene and alcohol gradients, respectively. Microwave-assisted antigen retrieval was performed in 1× buffer. To minimize background noise, endogenous peroxidase was inhibited with H_2_O_2_ and non-specific sites were blocked. Subsequently, the tissues were stained using primary antibodies against CD8 (clone 66868-1-Ig, 1:2000) and CX3CR1 (clone ab167571, 1:50). Subsequently, detection was achieved using HRP-conjugated secondary antibodies paired with tyramide signal amplification (TSA) fluorophores. Between staining rounds, antibody–TSA complexes were stripped using microwave heat treatment. Nuclei were counterstained with DAPI (4′,6-diamidino-2-phenylindole), and the slides were subsequently mounted using an antifade reagent. Whole-slide images were acquired using the ZEN 3.3 imaging system, and quantitative analysis of marker expression was performed using HALO pathology analysis software.

### Image acquisition and quantitative analysis

Whole-slide digital scanning of the stained TMAs was performed using the Zeiss Axioscan 7 system. Raw multispectral images were processed and analyzed using ZEN 3.3 software. Spectral unmixing was subsequently employed to differentiate individual fluorophore emissions. This process relied on a unique spectral signature library, which was meticulously established using singly stained tissue sections as controls. For quantitative analysis, the DAPI channel served as the nuclear mask to identify individual cells. Valid single cells were segmented by filtering based on nuclear area and fluorescence intensity, thereby excluding cellular debris and overlapping aggregates. Protein expression levels within specific cellular compartments (nuclear and/or cytoplasmic) were subsequently quantified. Specific intensity thresholds were established to define marker-positive populations, and the frequency of each cell subset was calculated relative to the total number of valid DAPI-stained cells.

### Public dataset acquisition and bioinformatics analysis

Human colorectal cancer scRNA-seq data were acquired from the GEO repository (GSE236581). The Seurat R package was employed for all subsequent analyses, including dimensionality reduction techniques and unsupervised clustering algorithms. Immune cell subsets, with a specific focus on CD8^+^T cells, were annotated based on canonical markers referenced in CellMarker 2.0 and visualized using ggplot2. To validate the clinical relevance and broad applicability of our findings, we curated a comprehensive compendium of bulk transcriptomic datasets. This included the TCGA-COAD cohort and multiple independent clinical cohorts of patients treated with ICIs (anti-PD-1/L1 or anti-CTLA-4). Multiple solid tumor cohorts were analyzed, spanning melanoma, urothelial carcinoma, renal cell carcinoma, and NSCLC, using data from IMvigor210 and GEO datasets (GSE78220, GSE91061, and GSE126044).

### Mice and cell lines

Female C57BL/6J mice, aged 6–8 weeks, were purchased from Cavens Laboratory Animal Co. and maintained under SPF conditions. Animal experiments were carried out following approval from the Ethics Committee of the Third Affiliated Hospital of Soochow University and in strict accordance with institutional regulations. MC38 cells, a mouse-derived colon adenocarcinoma line, were purchased from the Chinese Academy of Sciences (Shanghai, China) and maintained in DMEM containing 10% FBS and antibiotics at 37 °C in a humidified atmosphere with 5% CO_2_.

### *In vivo* tumor models and treatment

For the generation of the subcutaneous tumor model, C57BL/6J mice received a subcutaneous injection of MC38 cells (1×10^6^) suspended in 100 μL of PBS. Upon palpable tumor formation (Day 5 post-inoculation), mice were randomized into control and anti-PD-1 treatment cohorts. The treatment group received intraperitoneal (i.p.) injections of anti-mouse PD-1 monoclonal antibody (200 μ g/dose; Clone J43, Bio X Cell) on days 5, 8, 11, and 14. Control mice were administered matched isotype IgG. For downstream analyses, including flow cytometry and scRNA-seq, tumors were harvested 24 hours following the treatment.

### Flow cytometry and intracellular cytokine staining

To assess the phenotype and function of TILs, tumor specimens underwent enzymatic digestion with Liberase TL and DNase I (37 °C, 30 min). To prepare single-cell preparations, the cell mixture was passed through a 100-μm strainer, followed by suspension in Hank’s Balanced Salt Solution supplemented with 1% fetal calf serum. Lymphocytes were activated ex vivo using a Cell Activation Cocktail containing Brefeldin A (BioLegend) for 5 hours prior to staining to assess functional activity. For immunophenotypic characterization, surface and intracellular markers were assessed using a multistep flow cytometry protocol. Live cells were identified using a fixable viability dye (Ghost Dye™ Violet 510) and stained for CD45 (30F11), CD3 (17A2), CD4 (GK1.5), CD8 (53-6.7), and CX3CR1 (SA011F11). After fixation and permeabilization (Invitrogen), intracellular detection of interferon-γ (IFN-γ) (XMG1.2), tumor necrosis factor-alpha (TNF-α) (MP6-XT22), and Granzyme B (GB11) was performed. Data were collected on a DxFLEX system (Beckman Coulter) and processed using FlowJo software.

### Processing of mouse scRNA-seq data

Following a previously established protocol (37), alignment of raw reads to the mouse genome (mm10) was performed with the Cell Ranger software suite (v4.0.0). Subsequent downstream analyses were executed within the R environment using the Seurat package (v4.x). To ensure data integrity, a rigorous quality control pipeline was applied: potential doublets were excluded via DoubletFinder, and low-quality cells were filtered based on specific criteria (200 < nFeature_RNA < 6,000; nCount_RNA < 50,000; mitochondrial content < 10%). Principal Component Analysis was utilized to reduce data dimensionality linearly, following the application of LogNormalize scaling and the detection of HVGs. To mitigate technical batch effects across samples, the Harmony algorithm was employed for data integration. Using informative principal components, we applied non-linear embedding and unsupervised graph-based clustering implemented in FindNeighbors and FindClusters, after which cell types were annotated according to characteristic marker gene signatures.

### Pseudotime trajectory inference

To reconstruct the developmental hierarchy and differentiation lineages of CD8^+^T cells, trajectory inference was performed utilizing the Monocle 2 and Slingshot R packages. To facilitate Monocle analysis, gene expression matrices were extracted from the Seurat object and transformed into the required CellDataSet format. Statistical modeling of gene expression variance was conducted using the estimateSizeFactors and estimateDispersions functions. Dimensionality reduction and cell ordering were subsequently executed via the DDRTree (Discriminative Dimensionality Reduction via Learning a Tree) algorithm, based on the identified highly variable genes (HVGs). Cells were projected onto the inferred manifold to visualize the phenotypic transition from a naive state to an effector state along the latent pseudotime.

### Functional enrichment analysis

To elucidate the biological processes and molecular drivers responsible for the TME remodeling induced by anti-PD-1 therapy, we conducted systematic functional enrichment profiling of the identified differentially expressed genes (DEGs). The clusterProfiler package (version 4.8.3) was employed to assess functional enrichment across Gene Ontology domains and KEGG pathways. Annotation of mouse genes was based on the org.Mm.eg.db database. To account for multiple comparisons, *P*-values were corrected using the Benjamini–Hochberg approach, and enrichment results with FDR-adjusted *P*-values below 0.05 were retained. The most pertinent pathways upregulated or downregulated in the treatment group relative to controls were visualized via bar charts and bubble plots to illustrate the key functional transitions induced by PD-1 blockade.

### Cell-cell communication analysis

To characterize the landscape of intercellular communication, we employed the CellChat R package (version 1.6.1). Ligand–receptor interactions were systematically compared between the pre- and post-treatment cohorts utilizing the CellChatDB reference database. To ensure the robustness of our statistical inferences, interaction probabilities were estimated only for cell clusters containing at least 10 cells. Differential analysis was subsequently conducted to pinpoint signaling pathways exhibiting significant shifts in global information flow and cumulative interaction strength. Data visualizations were generated through the integrated functions of CellChat and the ComplexHeatmap (version 2.22.0) framework.

### Statistical analysis

All analyses were carried out in R (version 4.4.1), with figures generated using GraphPad Prism 8. The survminer package was applied to determine optimal thresholds for dichotomizing gene expression. Continuous data were compared between groups using the Wilcoxon test. The prognostic relevance of CX3CR1^+^CD8^+^T cell infiltration was assessed by Kaplan–Meier survival estimation and log-rank comparison. To construct a stable prognostic model, variables were screened through univariate Cox regression followed by Best Subset Regression (BSR) and LASSO regression with cross-validation. Factors retained from these analyses were subsequently included in a multivariate Cox proportional hazards model to identify independent prognostic determinants and develop a nomogram. A two-tailed P-value below 0.05 was considered statistically significant.

## Result

### Elevated levels of tumor-infiltrating CX3CR1^+^CD8^+^T cells predict favorable clinical outcomes

**Figure 1:**
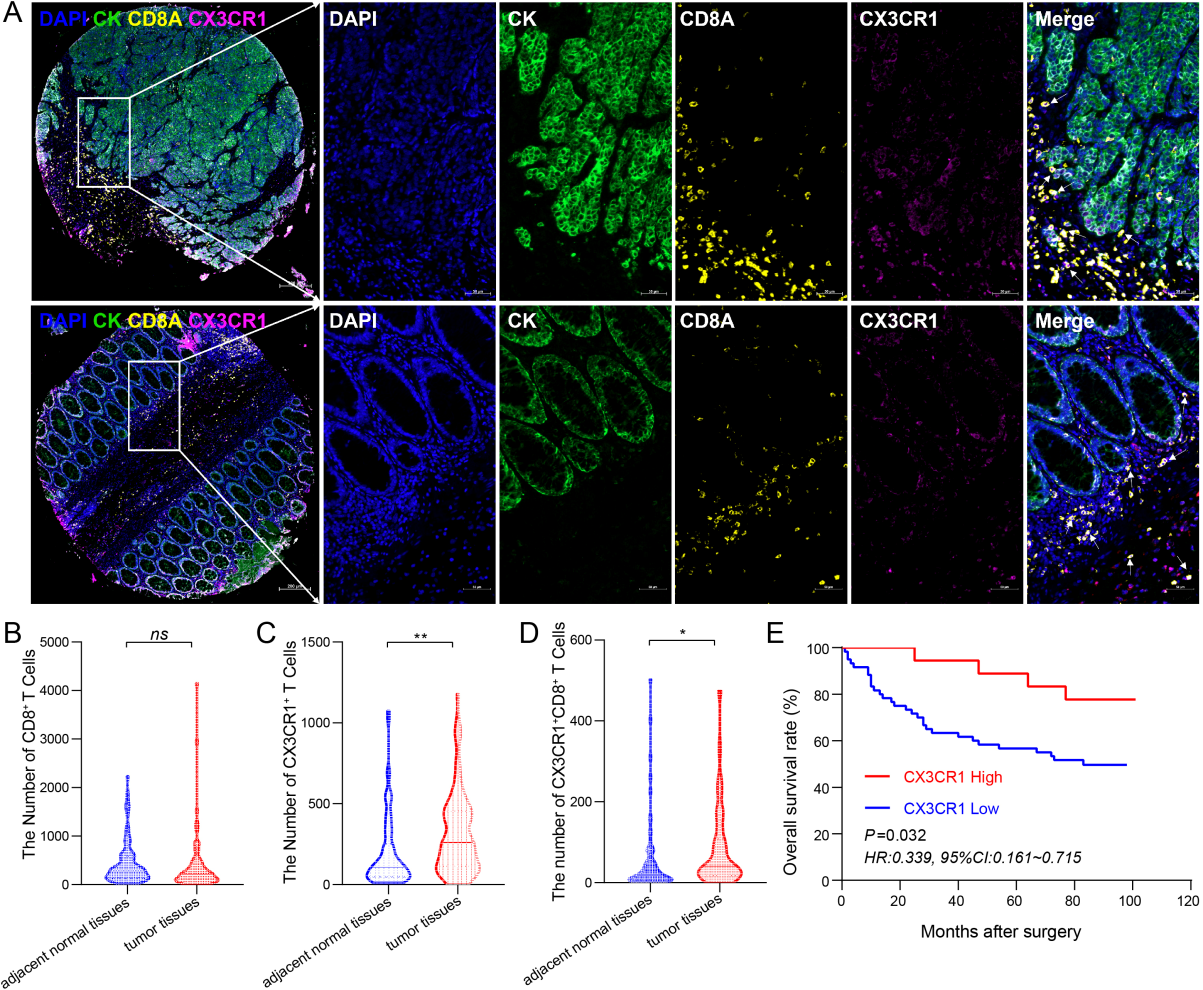
Identification and prognostic significance of CX3CR1^+^CD8^+^T cell infiltration in colorectal cancer. **(A)** Representative multiplex immunofluorescence images of tumor tissues (top row) and adjacent normal tissues (bottom row). The tissues were stained for DAPI (blue), Cytokeratin (CK, green), CD8A (yellow), and CX3CR1 (magenta). The white arrows in the merged panels indicate double-positive CX3CR1^+^CD8^+^T cells. Scale bars: 50 μm (200 μm for the whole slide view). **(B–D)** Quantitative comparison of immune cell infiltration densities between adjacent normal tissues and tumor tissues. The violin plots show the number of total CD8^+^ T cells [**(B)**, *ns*, not significant], total CX3CR1^+^ T cells [**(C)**, ^**^P < 0.01], and CX3CR1^+^CD8^+^ T cells [**(D)**, ^*^P < 0.05]. The results indicate a significant enrichment of CX3CR1^+^ and CX3CR1^+^CD8^+^T cell subsets in the tumor microenvironment. **(E)** Kaplan–Meier survival analysis of OS based on CX3CR1 expression levels. Patients with high CX3CR1 expression exhibited significantly better survival outcomes compared to the low expression group (Log-rank *P* = 0.032; *HR* = 0.339), suggesting its potential as a favorable prognostic marker.

To spatially characterize the infiltration pattern of CX3CR1^+^CD8^+^T cells and its clinical significance, mIHC was conducted on CRC specimens and their corresponding adjacent normal tissues. The infiltration of CX3CR1^+^CD8^+^T cells was evident in both stromal and intratumoral compartments, as verified by the precise co-localization of CD8A and CX3CR1 in representative images (**Figure 1A**). While overall CD8^+^T cell abundance was comparable between tumor and adjacent normal regions, CX3CR1^+^CD8^+^T cells were preferentially accumulated in the tumor microenvironment (P < 0.05; **Figures 1B–D**). Subsequently, we evaluated the prognostic significance of this specific subpopulation within a cohort of CRC patients. Patients exhibiting elevated infiltration of CX3CR1^+^CD8^+^T cells showed markedly improved OS relative to patients with lower infiltration, as determined by Kaplan–Meier analysis (*P* = 0.032; HR = 0.339, 95% CI: 0.161–0.715; **Figure 1E**). These findings suggest that the enrichment of CX3CR1^+^CD8^+^T cells in tumors is a powerful indicator of anti-tumor immunity and predicts good clinical outcomes.

### Construction and validation of a robust prognostic nomogram integrating the CX3CR1^+^CD8^+^T cell infiltration signature

This study assessed the clinicopathological correlation in 78 patients with CRC. Statistical evaluation revealed that the intratumoral abundance of CX3CR1^+^CD8^+^T cells was not significantly associated with patient age, gender, or TNM staging (*P* > 0.05). However, a marginally significant trend was observed regarding pathological grade (*P* = 0.081). Moreover, no significant association was observed between the abundance of total CD8^+^T cells or CX3CR1^+^ cells and the majority of clinicopathological parameters (**Table 1**). The lack of strong correlation between CX3CR1^+^CD8^+^T cells and traditional anatomical staging parameters suggests that the infiltration of this cell group represents an independent biological feature of the tumor immune microenvironment and can provide complementary information to existing staging systems.

**Table 1:** The correlations between CX3CR1^+^ cells, CD8^+^T cells, CX3CR1^+^CD8^+^T cells in tumor tissues and clinical features of patients with CRC.

Clinical parameter	Cases	Number of infiltrating CD8^+^T cells		*χ* ^2^	*P*	Number of infiltrating CX3CR1^+^ cells			*χ* ^2^	*P*	Number of CX3CR1^+^ CD8^+^T cells		*χ* ^2^	*P*
		Low	High			Low		High			Low	High		
Gender														
Male	37	28	9	0.902	0.342		30	7	0.045	0.832	30	7	0.686	0.408
Female	41	27	14				34	7			30	11		
Age (years)														
< 60	26	21	5	1.973	0.160		21	5	0.000	1.000^a^	21	5	0.325	0.569
≥ 60	52	34	18				43	9			39	13		
T stage														
T_2_	6	4	2	0.000	1.000^a^		5	1	0.000	1.000^a^	4	2	0.014	0.907^a^
T_3_+T_4_	72	51	21				59	13			56	16		
N stage														
N_0_	46	29	17	3.009	0.083		37	9	0.199	0.656	34	12	0.572	0.449
N_1_+N_2_	32	26	6				27	5			26	6		
M stage														
M_0_	75	52	23		0.345^b^		61	14		0.548^b^	57	18		0.450^b^
M_1_	3	3	0				3	0			3	0		
Pathological grade														
I/II/I–II	56	41	15	0.697	0.404		48	8	1.035	0.309	46	10	3.047	0.081
III/I–III/II–III	22	14	8				16	6			14	8		

^a^Continuity Adj. Chi-Square. ^b^Fisher’ Exact Test.

**Figure 2:**
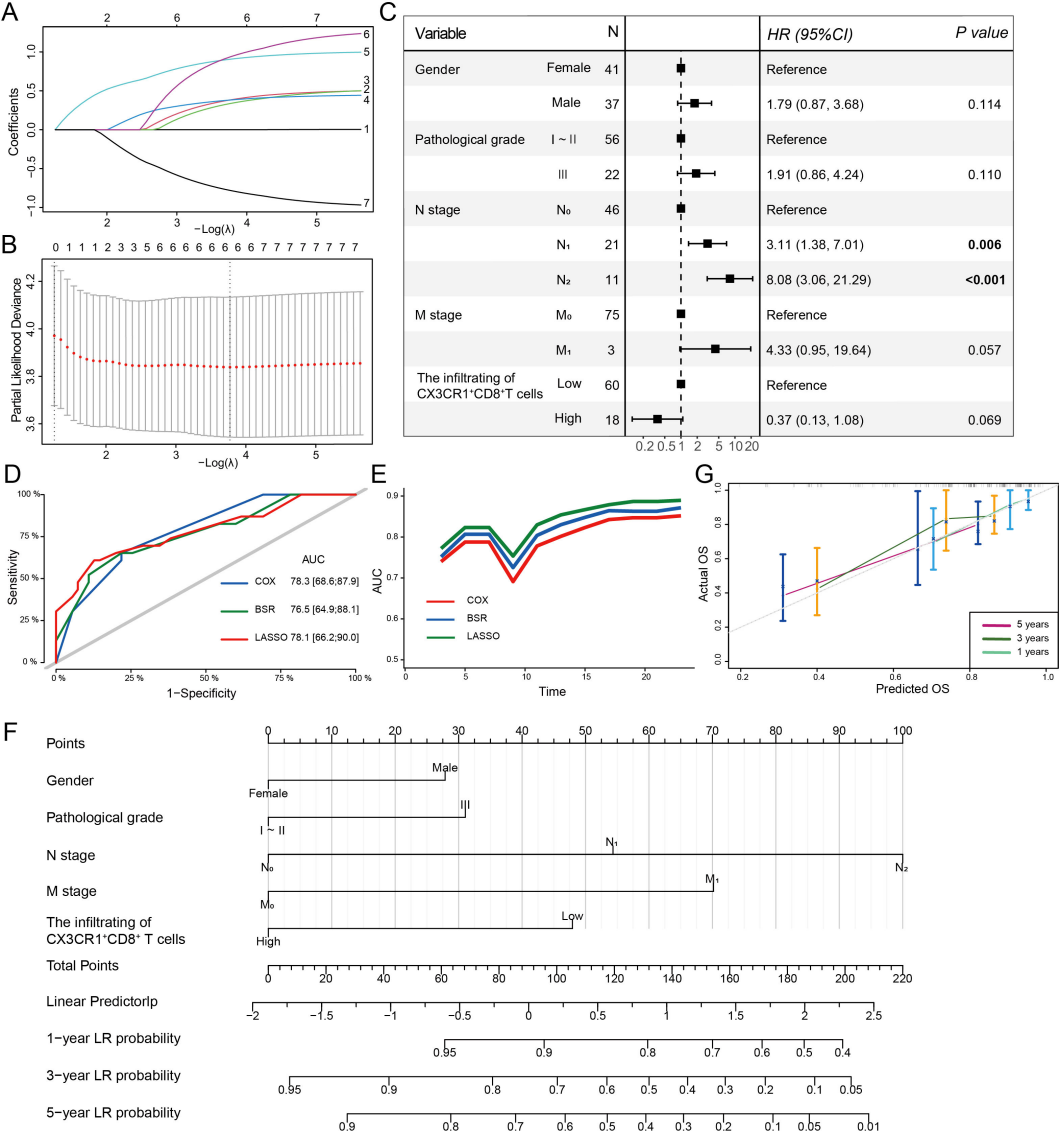
Construction and validation of a prognostic model incorporating CX3CR1^+^CD8^+^T cell infiltration. **(A)** LASSO coefficient profiles of the candidate prognostic features. Each colored curve represents the coefficient of a specific variable, illustrating how the coefficients shrink toward zero as the penalty parameter (λ) changes. **(B)** Selection of the optimal tuning parameter (λ) via 10-fold cross-validation. The y-axis represents the partial likelihood deviance, and the x-axis represents the log-transformed λ. The two vertical dotted lines indicate the optimal values determined by the minimum criteria (λ_min_, left) and the 1-standard error criteria (λ_1se_, right). **(C)** Forest plot illustrating the multivariate Cox regression model based on LASSO-selected variables. HR and 95% CI are displayed. **(D)** Receiver operating characteristic (ROC) curves comparing the predictive performance of Cox, BSR and LASSO models. **(E)** Time-dependent AUC curves evaluating the dynamic predictive accuracy over the follow-up period. **(F)** Nomogram for predicting 1-, 3-, and 5-year OS in CRC patients. **(G)** Calibration plots assessing the concordance between nomogram-predicted probabilities and observed survival. The gray diagonal line represents ideal prediction.

We implemented a multi-step statistical strategy to investigate the prognostic relevance of CX3CR1^+^CD8^+^T cell infiltration and to rigorously screen associated clinicopathological factors. In univariate Cox regression, advanced N stage emerged as a significant risk factor for survival (N1: HR = 2.496, *P* = 0.023; N2: HR = 6.333, *P* < 0.001), whereas higher levels of CX3CR1^+^CD8^+^T cell infiltration were linked to a protective effect (HR = 0.337, 95% CI: 0.119–0.957, *P* = 0.041) (**Table 2**; **Supplementary Figure 1A**). To mitigate selection bias and minimize the risk of overfitting, we employed the BSR algorithm, prioritized by the adjusted R^2^ metric, in conjunction with LASSO Cox regression. Both algorithms screened N stage and CX3CR1^+^CD8^+^T cell infiltration level as key variables; the LASSO model also included M stage (**Supplementary Figure 1B**; **Figures 2A–C**). Multivariate Cox regression analysis further confirmed that the protective role of high CX3CR1^+^CD8^+^T cell infiltration remained robust, even after controlling for potent clinical predictors such as N and M stages. (HR = 0.37 and 0.40 respectively) (**Supplementary Figure 1C**; **Figure 2C**). Although limited by the sample size of the high-infiltration subgroup (n = 18), its *P*-value was marginally significant (*P* = 0.069 and 0.09 respectively), but the significant effect size confirmed the potential of this subgroup as a robust prognostic biomarker independent of tumor metastasis status.

**Table 2:** Univariate analysis of prognostic factors for CRC patients.

Clinical parameters		Univariate analysis	
		HR (95% CI)	*P* value
Gender	Female	Reference	
	Male	0.994 (0.968–1.02)	0.633
Age (years)	< 60	Reference	
	≥ 60	1.145 (0.585–2.244)	0.692
Pathological grade	I–II	Reference	
	III	1.241 (0.593–2.596)	0.567
T stage	T_2_	Reference	
	T_3_	2.646 (0.342–20.506)	0.352
	T_4_	4.409 (0.594–32.746)	0.147
N stage	N_0_	Reference	
	N_1_	2.496 (1.137–5.476)	**0.023**
	N_2_	6.333 (2.653–15.116)	**0.000**
M stage	M_0_	Reference	
	M_1_	2.756 (0.658–11.539)	0.165
The infiltration of CX3CR1^+^CD8^+^T cells	Low	Reference	
	High	0.337 (0.119–0.957)	**0.041**

Bold italic signifies *P*<0.05.

We evaluated the discriminative power of different modeling approaches. The multivariate Cox model yielded the highest Area Under the Curve (AUC) of 78.3 (95% CI: 68.6–87.9), slightly outperforming the LASSO model (AUC = 78.1, 95% CI: 66.2–90.0) and the BSR model (AUC = 76.5, 95% CI: 64.9–88.1) (**Figure 2D**). However, considering the robustness of LASSO regression in minimizing overfitting and the high clinical relevance of the selected features, we prioritized the variables identified by LASSO to construct the final visual prognostic nomogram. This nomogram integrated N stage, M stage, gender, pathological grade, and CX3CR1^+^CD8^+^T cell infiltration. Notably, within this scoring system, low CX3CR1^+^CD8^+^T cell infiltration contributed significantly to the total risk score (approximately 48 points), visually quantifying the adverse prognostic impact of its absence (**Figure 2F**). Furthermore, time-dependent AUC analysis validated the reliability of the nomogram, demonstrating sustained predictive accuracy (AUC > 0.75) across the entire follow-up duration (**Figure 2E**). Furthermore, calibration plots exhibited a high degree of concordance between the predicted probabilities and the observed 1-, 3-, and 5-year OS rates (**Figure 2G**). In summary, the nomogram based on LASSO-selected features provides a precise and robust tool for the risk stratification of CRC patients.

### Single-cell profiling identifies the CX3CR1^+^CD8^+^T cell subset as a key responder population in PD-1 blockade-treated patients

**Figure 3:**
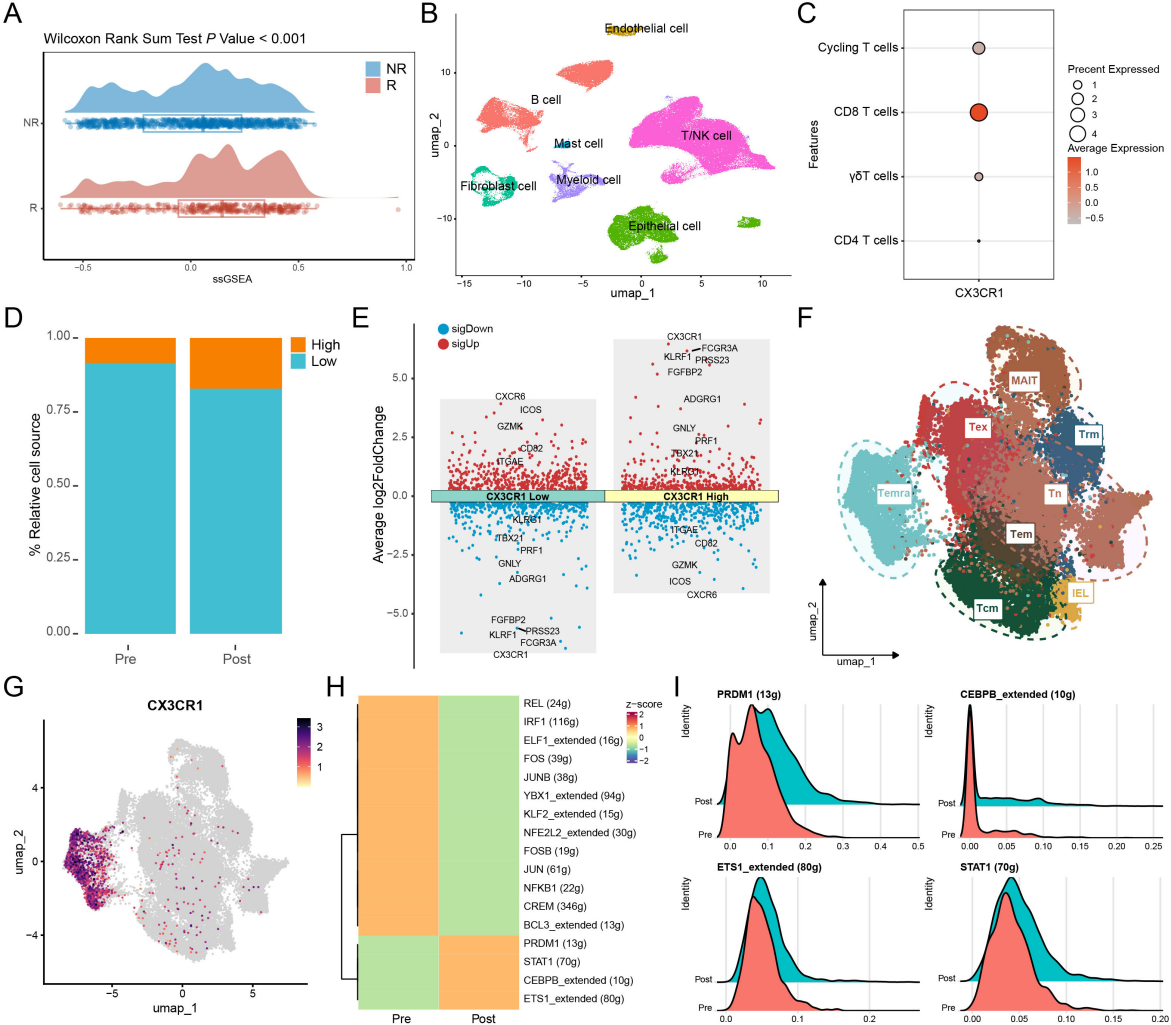
Transcriptional characterization and regulatory landscape of CX3CR1^+^CD8^+^T cells in PD-1 blockade responders. **(A)** Comparison of CX3CR1 signature scores (ssGSEA) between immunotherapy NR, non-responders and R, responders. **(B, C)** UMAP visualization of major cell lineages **(B)** and dot plot showing CX3CR1 expression specificity across T cell subsets **(C)** within the TME. **(D)** Relative proportions of CX3CR1^+^ and CX3CR1^−^CD8^+^T cells in paired pre- and post-treatment samples. **(E)** Mirror volcano plot depicting DEGs between CX3CR1^+^ and CX3CR1^−^ subsets. Red indicates upregulated cytotoxic/effector genes; blue indicates downregulated tissue-residency/exhaustion markers. **(F, G)** Sub-clustering of CD8^+^T cells visualized by UMAP **(F)** and feature plot showing CX3CR1 enrichment in the Temra cluster **(G)**. **(H)** SCENIC analysis of transcription factor regulon activity. Heatmap (left) displays differential activities between Pre- and Post-treatment groups (Z-score). **(I)** Ridge plots (right) visualize the activity distribution of *ETS1*, *PRDM1*, *STAT1*, and *CEBPB*. The x-axis represents the regulon activity level, and the height of the ridge represents the density of cells. Red/Salmon indicates the Pre-treatment group, and Teal indicates the post-treatment group.

To evaluate the predictive value of CX3CR1, we first analyzed its expression across multiple malignancies. Single-sample gene set enrichment analysis (ssGSEA) revealed that responders to anti-PD-1 therapy in melanoma, NSCLC and esophageal carcinoma exhibited significantly higher CX3CR1 scores than non-responders (*P* < 0.001; **Figure 3A**).

Focusing on the CRC TME, we re-analyzed a human scRNA-seq dataset. Unsupervised clustering identified major immune and stromal lineages (**Figure 3B**), with subsequent sub-clustering revealing that CX3CR1 expression was predominantly restricted to the CD8^+^T cell compartment (**Supplementary Figure 2A**; **Figure 3C**). Longitudinal analysis indicated a pronounced accumulation of CX3CR1^+^CD8^+^T cells within the TME of patients who exhibited a clinical response to anti-PD-1 therapy (**Figure 3D**). DGEs analysis characterized this subset as distinct from their CX3CR1^−^ counterparts, exhibiting upregulation of cytotoxicity (e.g., *GNLY*, *PRF1*, *FGFBP2*) and effector differentiation markers (e.g., *KLRG1*, *TBX21*). Conversely, genes associated with tissue residency (*ITGAE*, *CXCR6*) and precursor exhaustion (*GZMK*) were downregulated (**Figure 3E**). These features suggest that CX3CR1 marks a circulating CD8^+^T cell population with potent cytolytic potential, resembling a terminally differentiated effector memory (Temra) phenotype. High-resolution sub-clustering of CD8^+^T cells validated this identity, delineating distinct subsets including naive T cell (Tn), central memory T cell (Tcm), tissue-resident memory T cell (Trm), exhausted T cell (Tex), mucosal-associated invariant T cell (MAIT), intraepithelial lymphocytes (IEL), Tem and Temra (**Figure 3F**; **Supplementary Figure 2B**). Notably, robust CX3CR1 expression was specifically confined to the Temra cluster (**Figure 3G**), confirming its role as a marker for this effector subset.

**Figure 4:**
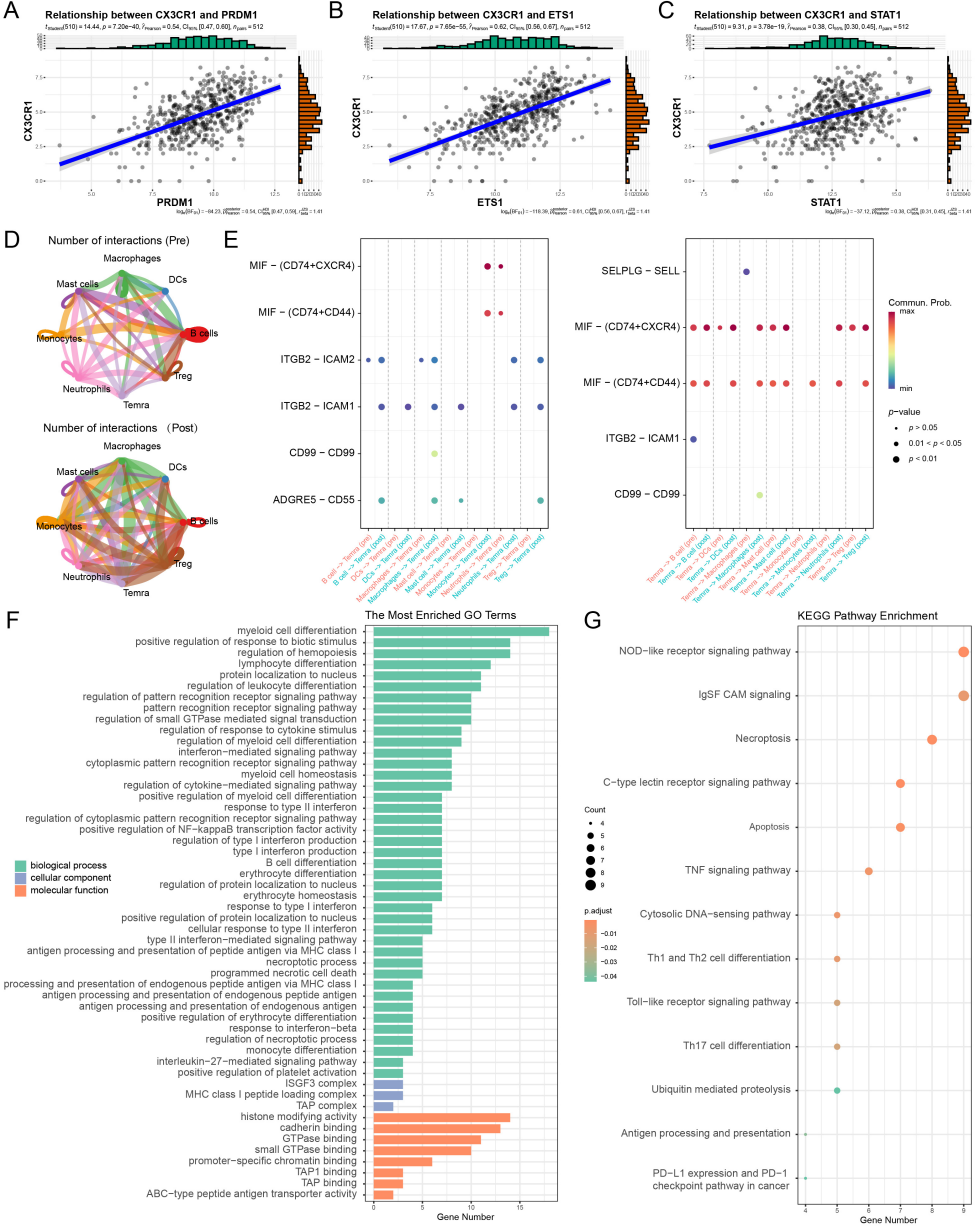
Validation of transcriptional regulators and functional profiling of the CX3CR1^+^ effector program. **(A–C)** Pearson correlation analysis between CX3CR1 expression and key transcription factors *PRDM1* **(A)**, *ETS1* **(B)**, and *STAT1* **(C)** in the TCGA-COAD cohort (n = 512). R indicates the Pearson correlation coefficient. **(D)** Comparative analysis of the inferred intercellular communication network topology between pre-treatment (top) and post-treatment (bottom) conditions. These circle plots reveal a fundamental reconfiguration of the immune crosstalk landscape, characterized by substantially augmented connectivity and signaling density involving Temra cells following therapy. **(E)** Bubble plots illustrating significant ligand-receptor interactions signaling toward CX3CR1^+^ Temra cells (Incoming, left) and originating from Temra cells (Outgoing, right). Dot colors denote the respective treatment groups where red represents pre-treatment and teal represents post-treatment. The dot size corresponds to the statistical significance of the inferred interaction. Note the striking enrichment of adhesion and migration-associated molecular axes, including ADGRE5–CD55, CD99–CD99, and SELPLG–SELL, within the post-treatment cohort. This pattern contrasts sharply with the dominance of MIF signaling observed in the pre-treatment group, indicating a therapeutic shift from a suppressive milieu toward an environment that favors effector cell recruitment. **(F, G)** Functional enrichment analyses of DEGs in the CX3CR1^+^ subset. **(F)** Top enriched GO terms categorized by BP, CC, and MF. **(G)** KEGG pathway enrichment analysis. Dot size represents gene count; color scale indicates adjusted *P*-value.

To elucidate the upstream mechanisms driving this phenotypic shift, we performed SCENIC analysis, which revealed a profound rewiring of the transcriptional landscape following immunotherapy. Baseline T cells were characterized by AP-1 family (e.g., *JUN*, *FOS*) and NFκB regulon activity, indicative of a stress-responsive state. In contrast, the post-treatment population exhibited a distinct regulatory program driven by effectors of differentiation and interferon signaling, notably *PRDM1* (encoding Blimp-1), *STAT1*, and *ETS1* (**Figure 3H**). Validating these findings in the TCGA-COAD cohort (n = 512), *CX3CR1* expression showed robust positive correlations with *ETS1* (*R* = 0.62, *P* < 0.001) and *PRDM1* (*R* = 0.54, *P* < 0.001), reinforcing their role in orchestrating the terminal effector program (**Figures 4A, B**). *CX3CR1* also correlated significantly with *STAT1* (*R* = 0.38, *P* < 0.001), consistent with interferon pathway activation (**Figure 4C**). While *CEBPB* was active at the single-cell level, its correlation in bulk tissue was non-significant (*P* > 0.05), likely due to confounding expression in stromal or myeloid compartments (**Supplementary Figure 2C**). Collectively, these data suggest an *ETS1*, *PRDM1* and *STAT1*-driven network regulates CX3CR1^+^CD8^+^T cell infiltration.

While intrinsic transcriptional reprogramming governs effector differentiation, the spatial accumulation of these cells is critically modulated by extrinsic signals within the TME. To determine how PD-1 blockade reshapes the intercellular communication landscape to facilitate this process, we performed CellChat analysis. At a global level, therapy significantly intensified immune crosstalk, as evidenced by a marked increase in both the frequency and cumulative strength of inferred ligand-receptor interactions in post-treatment samples (**Figure 4D**; **Supplementary Figures 2D, E**). Differential interaction analysis further revealed that this signaling enhancement was preferentially concentrated between Temra cells and myeloid-derived antigen-presenting cells (APCs). A comparative analysis of signaling flow identified a profound transition in the TME landscape, where the MIF pathway, a known mediator of immune suppression at baseline, became markedly attenuated following therapy (**Figure 4E**). Conversely, pathways essential for leukocyte adhesion and transendothelial migration were robustly up-regulated post-treatment. Specifically, Temra cells received intensified incoming signals through the ADGRE5 (CD97) and CD99 axes from myeloid cells while simultaneously engaging the TME via outgoing SELPLG interactions (**Figure 4E**; **Supplementary Figure 2F**). Collectively, these findings indicate that PD-1 blockade orchestrates a more permissive immune niche by dismantling inhibitory barriers while reinforcing the physical docking mechanisms required for successful Temra engraftment.

Complementing these regulatory and communicative alterations, functional enrichment analyses of the DEGs provided further insights into the biological programming of CX3CR1^+^CD8^+^T cells. GO analysis revealed a predominant enrichment in pathways governing lymphocyte differentiation and interferon-β/γ responses (**Figure 4F**), underscoring a robust commitment to effector functions. Notably, the upregulation of antigen processing machinery (e.g., TAP binding) suggests enhanced tumor recognition capabilities, while the enrichment of histone-modifying activities implies that epigenetic remodeling underpins this terminal phenotype. Corroborating these findings, KEGG pathway analysis highlighted the activation of inflammatory and cytotoxic signaling, including TNF, necroptosis, and apoptosis pathways (**Figure 4G**). Furthermore, the specific enrichment of the PD-1/PD-L1 checkpoint pathway reinforces the clinical relevance of this subset in the context of ICIs. Collectively, these signatures define CX3CR1^+^CD8^+^T cells as a highly activated, epigenetically distinct, and functionally potent effector population.

### PD-1 blockade reinvigorates antitumor immunity by specifically promoting the expansion and functional maintenance of CX3CR1^+^CD8^+^T cell

**Figure 5:**
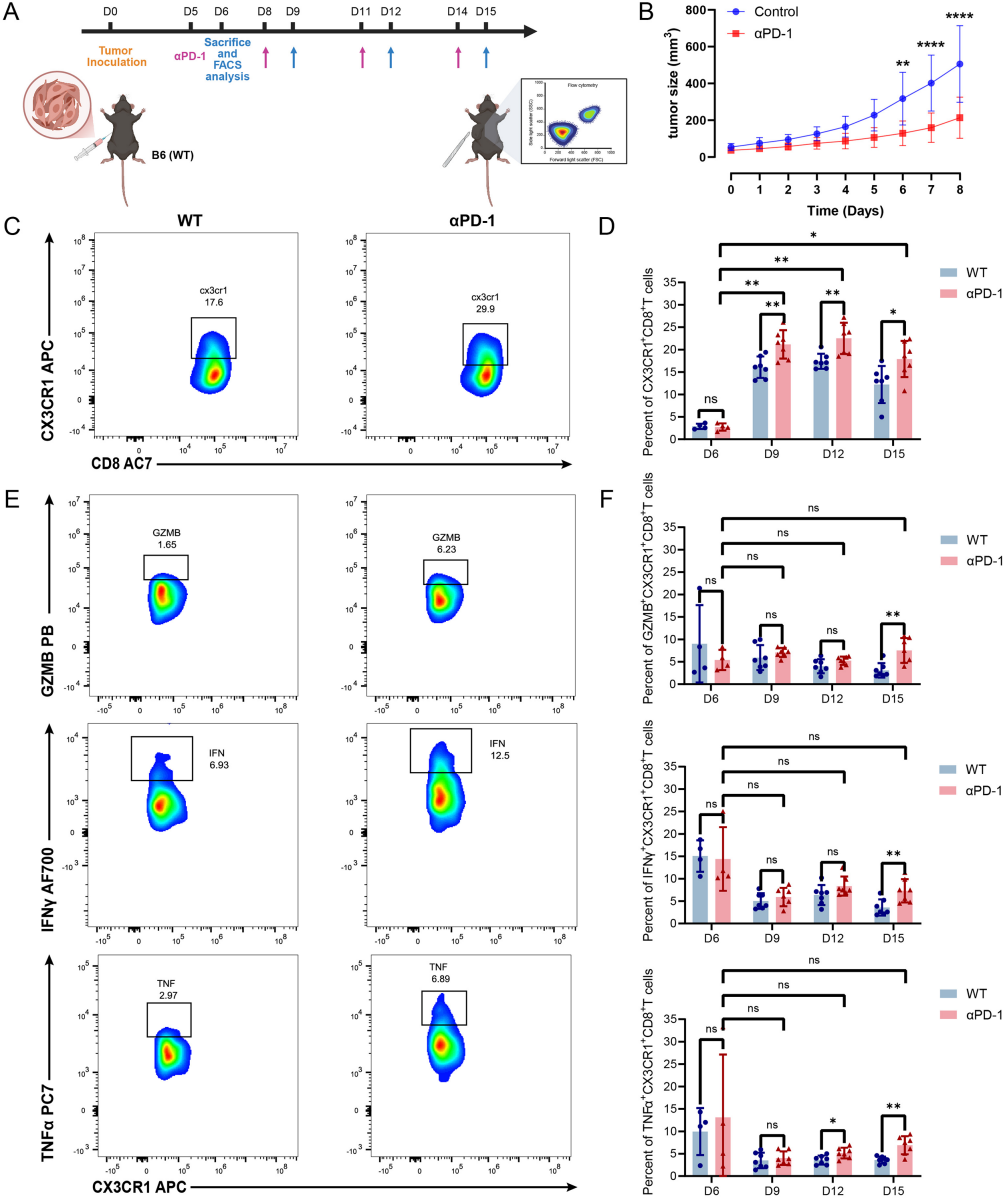
Anti-PD-1 therapy promotes the expansion and functional maintenance of intratumoral CX3CR1^+^CD8^+^T cells. **(A)** Timeline of anti-PD-1 administration and tissue collection in C57BL/6J mice. **(B)** Monitoring of tumor progression over time. **(C, E)** Typical FACS plots highlighting the accumulation of CX3CR1^+^CD8^+^T cells **(C)** and their production of cytotoxic/inflammatory mediators (GZMB, IFN-γ, TNF-α) **(E)**. **(D, F)** Comparative analysis of the abundance of the CX3CR1^+^ subset **(D)** and the proportions of functional markers within this specific population **(F)**. Error bars indicate SEM (n = 7). ^*^P < 0.05, ^**^P < 0.01, ^****^P < 0.0001 (two-way ANOVA).

To evaluate the therapeutic efficacy of immune checkpoint blockade, MC38 tumor-bearing C57BL/6J mice were administered PD-1 antibodies beginning five days after tumor inoculation. As shown in the figures (**Figures 5A, B**; **Supplementary Figure 3A**), PD-1 blockade significantly attenuated tumor progression compared to controls, with therapeutic benefits becoming statistically significant after just two doses (*P* < 0.01) and intensifying over time. Having confirmed the model’s responsiveness, we next interrogated the longitudinal kinetics of CX3CR1^+^CD8^+^T cell subsets within the tumor microenvironment. Flow cytometry data demonstrated that the treatment induced a marked and selective accumulation of the CX3CR1^+^CD8^+^T cell population. Notably, this enrichment was spatially confined to the tumor tissue, while it was absent in the spleen and lymph nodes, and became prominent starting from day 9 post-inoculation (**Figures 5C, D**; **Supplementary Figures 3B, C**), suggesting a localized reinvigorating effect on this specific effector population.

Subsequently, we examined the capacity of anti-PD-1 therapy to preserve the functional status of the CX3CR1^+^CD8^+^T cell population throughout tumor development. Longitudinal profiling revealed that in control group, intratumoral CX3CR1^+^CD8^+^T cells underwent a progressive loss of functional potency, evidenced by diminished expression of Granzyme B (GZMB), IFN-γ and TNF-α by day 15. In contrast, anti-PD-1 therapy effectively counteracted this functional decay. The treatment group maintained robust expression of GZMB and preserved polyfunctional capacity, with significantly higher proportions of IFN-γ- and TNF-α-producing cells at the late tumor stage compared to controls (**Figures 5E, F**). Additionally, a transient but distinct peak in effector function was observed in secondary lymphoid organs (spleen and lymph nodes) around day 9, pointing to a systemic priming event that may precede the sustained response within the tumor (**Supplementary Figures 3B, C**). Taken together, our findings demonstrate that PD-1 blockade sustains the potent cytotoxic function of CX3CR1^+^CD8^+^T cells, effectively averting the functional decline that typically accompanies tumor growth.

### Transcriptional reprogramming toward a hyper-metabolic and migratory Temra phenotype drives the PD-1-induced T cell response

**Figure 6:**
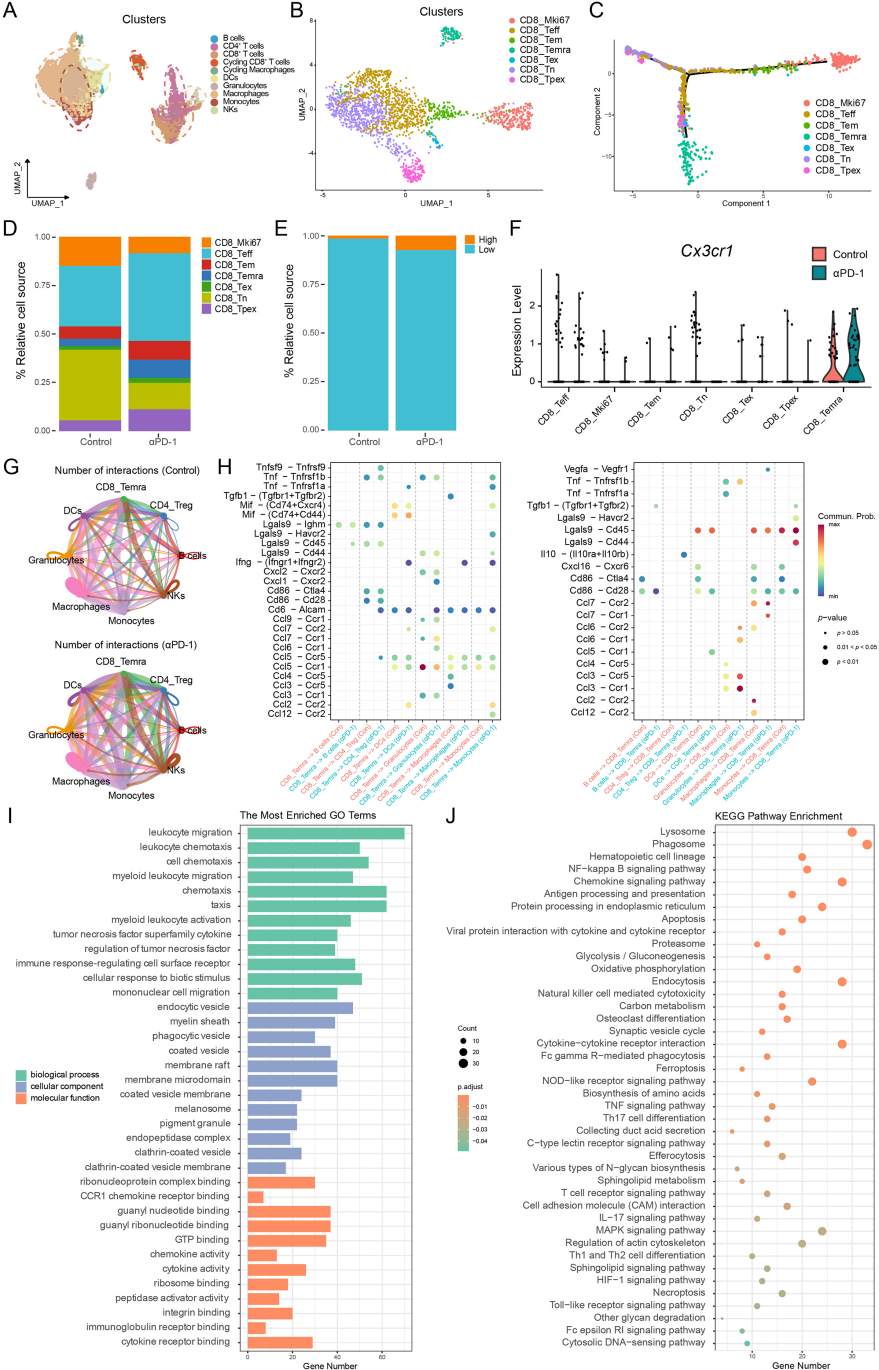
Single-cell transcriptomic remodeling of the CX3CR1^+^CD8^+^T cell compartment by PD-1 blockade. **(A, B)** UMAP visualization of the TME **(A)** and sub-clustering of CD8^+^T cells **(B)**, identifying distinct subsets including Tn, Teff, Tpex, Tex, Tem, Mki67 CD8^+^T cells and Temra cells. **(C)** Pseudotime differentiation trajectory analysis of CD8^+^T cell subsets. **(D)** Stacked bar charts showing the relative proportions of each CD8^+^T cell subset in the Control and anti-PD-1 treatment groups. **(E)** Bar plot quantifying the relative proportion of cells with high versus low expression of the signature of interest (e.g., *Cx3cr1*) between groups. **(F)** Violin plots illustrating *Cx3cr1* expression levels across CD8^+^T cell subsets stratified by treatment group. **(G)** Topological comparison of intercellular communication networks in control (top) versus αPD-1 (bottom) cohorts. Edge thickness within the circle plots reflects the relative strength of inferred interactions. Following PD-1 blockade, a marked expansion in the communication network is evident, with Temra cells emerging as a central signaling hub through substantially increased connectivity with the surrounding tumor microenvironment. **(H)** Analysis of specific signaling dynamics as either outgoing (left) or incoming (right) interactions for the CD8^+^Temra subset. In response to αPD-1 therapy, Temra cells exhibit enhanced effector output through *Ccl5* and *Ifng* pathways, while simultaneously receiving robust stimulatory and migratory cues via *Cd86*–*Cd28* and *Cxcl16*–*Cxcr6* signaling. The color scheme identifies the treatment cohorts (yellow/red for control; blue/teal for αPD-1), with the diameter of each dot representing the *P*-value. This reciprocal signaling enhancement suggests a more activated and precisely recruited effector T cell state following PD-1 blockade. **(I, J)** Functional enrichment analyses of DEGs between treatment groups. **(I)** Top enriched GO terms categorized by BP, CC, and MF. **(J)** KEGG pathway enrichment analysis indicating activated signaling and metabolic programs.

Using MC38 tumor-bearing mice treated with PD-1 inhibitors, we performed scRNA-seq to map the transcriptomic signature underlying the accumulation of CX3CR1^+^CD8^+^T cells (**Figure 6A**). Unsupervised clustering identified distinct CD8^+^T cell subsets, including naive T cell (Tn), effector T cell (Teff), progenitor exhausted T cell (Tpex), exhausted T cell (Tex), Tem, Ki67-positive (Mki67) CD8^+^T cell and Temra cell populations (**Figure 6B**; **Supplementary Figure 4A**). Quantitative analysis revealed a significant remodeling of the T cell compartment following PD-1 blockade, characterized by a contraction of naive and proliferating clusters and a concomitant expansion of effector-like subsets, particularly the Temra population (**Figure 6D**). To delineate the developmental hierarchy driving this shift, we performed single-cell pseudotime trajectory analysis. The trajectory revealed a continuous differentiation process rooting from Tn clusters, which bifurcated into two distinct terminal states (**Figure 6C**). While one branch progressed toward the classical Tex cluster, a divergent trajectory led to the formation of the Temra cluster. The Tpex subset occupied a critical transitional position preceding this bifurcation, suggesting its plasticity to differentiate into either lineage. This finding provides compelling evidence that the Temra population represents a lineage distinct from terminal exhaustion, retaining effector potential rather than succumbing to dysfunction. Notably, *Cx3cr1* expression was predominantly confined to this Temra subset and was significantly upregulated upon PD-1 blockade, corroborating our flow cytometry results and identifying CX3CR1 as a marker of this therapy-responsive effector population (**Figures 6E, F**).

DEGs analysis revealed that PD-1 blockade induced a distinct transcriptional signature characterized by the robust upregulation of chemokines (e.g., *Cxcl9, Ccl12, Ccl2*) and the co-stimulatory receptor *Tnfsf9*, alongside the metabolic regulator *Cd36*. In contrast, *Gzmk*, a canonical marker of precursor exhausted T cells, was significantly downregulated. This transcriptional shift suggests that PD-1 blockade effectively abrogates the differentiation block, propelling T cells from a transitional state toward a terminal effector phenotype (**Supplementary Figure 4B**).

To dissect the intercellular communication networks facilitating this differentiation and expansion, we performed CellChat analysis on the murine scRNA-seq dataset. PD-1 blockade markedly invigorated the TME interactome, as evidenced by a substantial increase in both the frequency and cumulative strength of cell-cell interactions (**Figure 6G**; **Supplementary Figure 4D**). Differential network analysis revealed that this functional enhancement was largely driven by intensified crosstalk between CD8^+^ Temra cells and myeloid populations including macrophages and dendritic cells (**Supplementary Figure 4C**).

Pathway analysis uncovered a profound switch in the signaling landscape, where control tumors were dominated by immunosuppressive axes such as *TGFβ* and *MIF*, whereas PD-1 blockade significantly upregulated pro-inflammatory and recruitment-associated pathways including *CXCL*, *CCL*, *IFN-II*, and *CD86* (**Supplementary Figure 4E**). Notably, we identified a bidirectional positive feedback loop where Temra cells actively secreted *IFN-γ* and chemokines such as *CCL5* to remodel the TME, while reciprocally receiving potent costimulatory signals via the *CD86*–*CD28* axis and retention cues via the *CXCL16*–*CXCR6* axis from antigen-presenting cells (**Figure 6H**).

Complementing this intercellular rewiring, we interrogated the functional landscape using GO and KEGG enrichment analyses (**Figures 6I, J**). GO enrichment analysis highlighted a significant activation of pathways related to leukocyte migration and chemotaxis. These gene expression results, consistent with the chemokine-receptor interactions identified by CellChat, clarify the molecular mechanisms driving the elevated intratumoral infiltration of CX3CR1^+^CD8^+^T cells observed via flow cytometry. Concomitantly, the upregulation of ribosome biogenesis and cytokine activity signatures indicates a hyper-metabolic state, essential for sustaining potent effector protein synthesis. Corroborating these observations, KEGG analysis highlighted the activation of pivotal immune axes, including chemokine, T-cell receptor (TCR), and NF-κB signaling pathways. Notably, the significant enrichment of metabolic pathways, particularly oxidative phosphorylation, implies that PD-1 blockade drives metabolic reprogramming to meet the high bioenergetic requirements of antitumor cytotoxicity. Furthermore, the prominence of lysosome-related pathways points to an augmented capacity for secretory granule processing and degranulation. Collectively, these data demonstrate that anti-PD-1 therapy reinvigorates antitumor immunity by orchestrating a multifaceted program that integrates enhanced recruitment, metabolic fitness, and cytotoxic effector function.

## Discussion

In the era of ICIs, the quantity and quality of TILs are fundamental determinants of therapeutic efficacy (38, 39). Although CD8^+^ CTLs serve as the central mediators of antitumor immune responses, clinical efficacy is fundamentally governed by their functional diversity, which ranges from stem-like precursor states to terminal exhaustion (40). Herein, we comprehensively profiled a unique subpopulation of CX3CR1^+^CD8^+^T cells within the context of CRC. Contrary to the traditional paradigm equating terminal differentiation with functional exhaustion, our multi-omics analysis identified these cells as a non-exhausted, highly cytotoxic effector memory population (Temra). Clinically, high infiltration of this subset served as a robust independent prognostic factor, offering superior risk stratification compared to traditional TNM staging. These findings align with emerging evidence in melanoma and NSCLC, linking CX3CR1^+^ effectors to enhanced ICIs responsiveness (31, 33).

In addition to its general prognostic value, the enrichment of CX3CR1^+^CD8^+^T cells is particularly relevant to the unique histopathological features of Lynch syndrome tumors and sporadic MSI-H colorectal cancers. These tumors are characterized by defects in mismatch repair proteins, leading to microsatellite instability and a consequent high tumor mutational burden (TMB) (41). The resulting abundance of frameshift peptide neoantigens creates a highly immunogenic microenvironment that robustly primes T cell responses. We propose that this neoantigen-rich context acts as a continuous driver for the differentiation of stem-like progenitors into the CX3CR1^+^CD8^+^T cell. Unlike in low-mutation MSS tumors where T cell priming may be suboptimal, the strong antigenic signal in Lynch syndrome tumors, especially when unleashed by PD-1 blockade, likely fuels the sustained accumulation of these cytotoxic CX3CR1^+^ effectors, thereby contributing to the superior clinical outcomes and immunotherapy responsiveness typically observed in this patient population (42). However, due to the limited sample size of our scRNA-seq cohort, which was skewed toward MSI-H patients, we were unable to perform a robust comparative analysis of CX3CR1^+^CD8^+^T cell infiltration between MSI-H and MSS subtypes. Given the distinct immunogenic landscapes of these two subtypes, future studies with larger, balanced cohorts are needed to elucidate how different tumor microenvironments differentially regulate the recruitment and function of this effector subset. Additionally, a critical question remains regarding the utility of T cells in stratifying patients with Lynch syndrome or sporadic MSI-H tumors, who generally exhibit a favorable prognosis. While our current study focused on the general CRC population, we hypothesize that this subset could serve as a sensitive indicator of effective immune surveillance specifically within the MSI-H context. Although high tumor mutational burden in Lynch syndrome tumors provides the antigenic potential, it does not guarantee an effective immune response. The infiltration of effectors confirms the cytotoxic execution. Therefore, we propose that the paucity of this subset might identify the minority of Lynch syndrome patients at higher risk of recurrence (immune evasion despite high antigenicity), who would otherwise be overlooked by traditional staging. Future studies with larger, specifically designed MSI-H cohorts are warranted to statistically validate this stratification potential, which was limited in our current public dataset analysis due to the low event rate in this favorable-prognosis group.

A pivotal finding of our study is the specific expansion of the CX3CR1^+^ subset following PD-1 blockade. In our murine models, therapy did not merely increase total CD8^+^ infiltration but specifically enriched this effector population. Transcriptomically, these cells exhibited a unique “differentiation success” signature: downregulation of the precursor exhaustion marker GZMK and upregulation of cytotoxic molecules (GNLY, PRF1). This refines the current “stem-like rejuvenation” model (25) by highlighting that therapeutic benefit is ultimately mediated by the accumulation and functional persistence of these terminal effectors, rather than the progenitors themselves. Furthermore, the sustained expression of IFN-γ and TNF-α at late tumor stages indicates that PD-1 blockade effectively prevents the functional attrition typically driven by chronic antigen stimulation (24).

We further elucidated the mechanisms underlying this accumulation. The transcriptomic enrichment of leukocyte migration pathways, coupled with the upregulation of chemokines (CXCL9, CCL12) and their receptors, suggests that PD-1 blockade remodels the chemokine landscape to actively recruit these effectors from the circulation. This “peripheral recruitment” model challenges the view that intratumoral expansion relies solely on local proliferation (43) and is strongly supported by recent fate-mapping studies demonstrating that sustained antitumor immunity depends on the continuous replenishment of CX3CR1^+^ effectors from lymph node-resident progenitors (44). Beyond this initial recruitment driven by chemokine gradients, the persistence and functional execution of CX3CR1^+^CD8^+^T cells within the TME rely heavily on their dynamic crosstalk with neighboring immune populations. Our intercellular communication analysis reveals that PD-1 blockade orchestrates a multi-stage retention and activation program. Initially, the therapy-induced upregulation of adhesion axes, such as *ADGRE5* (*CD97*) and *CD99*, facilitates the physical anchorage of recruited effectors to myeloid antigen-presenting cells (APCs) and the stromal matrix. This docking mechanism is crucial for stabilizing the immune synapse and ensuring durable T cell engraftment. Subsequently, we observed a reinforcement of the *CD86*–*CD28* costimulatory axis and the establishment of a positive feedback loop mediated by *IFN-γ* and *CCL5*. This suggests that once retained, CX3CR1^+^CD8^+^T cells actively remodel the local niche to promote the maturation of DCs and macrophages which, in turn, reciprocally sustain T cell effector function. Furthermore, the marked attenuation of *MIF* and *TGF-β* signaling pathways post-treatment indicates a dismantling of the tolerogenic network that typically supports regulatory T cells (Tregs). Collectively, these findings suggest that the accumulation of CX3CR1^+^CD8^+^T cells is not merely a passive influx but the result of a coordinated ecological restructuring that favors effector retention over suppression.

Moreover, the functional persistence of these recruited cells faces a severe challenge: the hypoxic tumor microenvironment. Chronic hypoxia is a hallmark of CRC that typically stabilizes Hypoxia-inducible factor 1-alpha (HIF-1α) and represses mitochondrial biogenesis, driving T cells toward metabolic insufficiency and terminal exhaustion (17, 45). In this hostile context, the robust enrichment of oxidative phosphorylation and ribosome biogenesis pathways in the CX3CR1^+^CD8^+^T cell subset is particularly significant. It indicates that PD-1 blockade confers a critical metabolic fitness, allowing these effectors to maintain mitochondrial respiration and resist hypoxia-driven dysfunction. We propose that this metabolic resilience prevents the HIF-1α-mediated exhaustion program and enables CX3CR1^+^CD8^+^T cells to sustain the high bioenergetic demands of cytotoxic function, thereby setting them apart from metabolically exhausted populations (46). Consequently, combining PD-1 blockade with hypoxia-targeting agents (e.g., HIF inhibitors or oxygenation agents) represents a promising therapeutic strategy to further unleash the potential of this effector subset (47, 48).

At the transcriptional level, we identified an *ETS1-* and *PRDM1*-driven network governing this effector differentiation. SCENIC analysis revealed a dramatic shift from a pre-treatment AP-1-dominated stress response to a post-treatment effector program orchestrated by *ETS1*, *PRDM1*, and *STAT1*. While *ETS1* is classically associated with maintaining stemness in progenitor-exhausted cells (49), our data reveal a context-dependent role where *ETS1* is robustly upregulated in the terminally differentiated CX3CR1^+^ Temra subset following immunotherapy. We propose that in this therapeutic context, sustained *ETS1* activity synergizes with *PRDM1*, a master regulator of cytotoxicity (50), to drive a migratory yet cytotoxic program, thereby preventing the epigenetic silencing typically associated with terminal exhaustion.

Despite the mechanistic and clinical insights provided by this study, several limitations warrant further investigation. The current transcriptomic analysis relies on a relatively limited number of scRNA-seq samples, and the clinical validation, while statistically robust, is based on a retrospective, single-center cohort, which may introduce inherent selection bias. To firmly establish CX3CR1^+^CD8^+^T cell infiltration as a clinically actionable biomarker, particularly for stratifying the challenging MSS population, validation through large-scale, multi-center prospective studies is indispensable. Beyond clinical validation, higher-resolution mapping of the spatial dynamics and cellular origins of this subset is required. Although mIHC confirmed the localization of these cells within tumor nests, their precise interplay with antigen-presenting cells remains to be fully elucidated. For instance, given that dendritic cell-derived CX3CL1 orchestrates T cell recruitment in a circadian-dependent manner (51), future studies should determine whether CX3CR1^+^CD8^+^T cells rely on specific spatial niches, such as tertiary lymphoid structures (52), for their recruitment and maintenance. Concurrently, definitive lineage tracing or photoconversion models (26) are needed to distinguish whether the post-treatment expansion arises predominantly from the local proliferation of Tpex cells or the systemic recruitment of circulating effectors.

Moreover, the long-term implications of this effector differentiation warrant careful examination. Consistent with the ‘rheostat’ model (53), the acceleration of progenitor differentiation into effector states driven by PD-1 blockade may inadvertently compromise the longevity of the renewable stem-like reservoir. In our context, the marked accumulation of CX3CR1^+^CD8^+^T cells indicates the effective deployment of antitumor effectors. Conversely, in the context of acquired resistance, this phenomenon may signal an excessive depletion of the upstream Tpex reservoir, rendering the response unsustainable. Therefore, optimal therapeutic strategies must aim to balance the mobilization of CX3CR1^+^ effectors with the preservation of their progenitors to ensure durable tumor control. Ultimately, while we identified the *ETS1* and *PRDM1*-driven transcriptional network, the chromatin landscape regulating this phenotypic lock remains unexplored. Unraveling the epigenetic stability of this subpopulation could reveal novel targets to prevent dedifferentiation or premature exhaustion.

We recognize a seemingly contradictory report suggesting that CX3CR1^+^CD8^+^T cell depletion does not abrogate adoptive cell therapy (ACT) efficacy (54). This discrepancy likely reflects distinct mechanisms: ACT relies on the infused stem-like reservoir, whereas PD-1 blockade depends on reinvigorating the endogenous host immune system. In our experimental setting, the expansion of the CX3CR1^+^CD8^+^T cell compartment functions as a pivotal indicator reflecting effective differentiation and therapy-induced effector mobilization. The lack of efficacy in non-responders likely stems from a blockage in this differentiation trajectory. Furthermore, functional redundancy among chemokine receptors (e.g., CXCR3) may compensate for CX3CR1 loss in knockout models, a hypothesis warranting further investigation.

Finally, we acknowledge that our study relies on correlative analyses from human data and *in vivo* observational models. While we identified cells as a key effector subset, we did not perform T cell-specific *Cx3cr1* knockout or overexpression experiments. Therefore, we cannot definitively conclude that CX3CR1 itself is the driver of effector function, rather than a surface marker of a broader transcriptional program. Future studies utilizing conditional knockout models are needed to dissect the specific functional contribution of the CX3CR1 receptor versus its associated regulatory network.

In summary, our study establishes CX3CR1^+^CD8^+^T cells as a pivotal determinant of prognosis and immunotherapy responsiveness in colorectal cancer. Clinically, tracking the dynamic expansion of this subset offers a robust biomarker for refining risk stratification and monitoring therapeutic efficacy, particularly addressing the challenge of response heterogeneity within the MSI-H population. Mechanistically, we propose that therapeutic failure, including both primary non-response and acquired resistance, results from a differentiation block that impairs the generation or maintenance of this specific effector phenotype. By elucidating the transcriptional network orchestrated by *ETS1* and *PRDM1*, our findings provide a compelling rationale for next-generation therapies designed to mobilize this potent effector reservoir, thereby offering a novel strategy to circumvent immune resistance. 

## Data Availability

The human single-cell RNA sequencing data analyzed in this study are publicly available in the Gene Expression Omnibus (GEO) repository under accession number GSE236581, GSE78220, GSE91061, and GSE126044. The mouse sequencing datasets presented in this article are not readily available because they are part of ongoing research and may be used in future publications. Requests to access the datasets should be directed to the corresponding author at zhengxiao@suda.edu.cn.

## Supplementary material

Supplementary Figure 1 Identification of independent prognostic factors for CRC patients via multi-dimensional statistical approaches. **(A)** Forest plot of the multivariate Cox regression model incorporating significant univariate predictors. **(B)** Variable selection using BSR based on the adjusted R^2^ criterion. The optimal model with the highest adjusted R^2^ is identified. **(C)** Multivariate Cox regression model constructed using BSR-selected variables (Gender, N stage, and CX3CR1^+^CD8^+^T cell infiltration), confirming the protective value of the immune subset (HR = 0.40).

Supplementary Figure 2 Characterization of T cell subtypes and validation of CEBPB correlation in CRC. **(A)** UMAP visualization of the total T cell compartment isolated from tumor tissues, resolving major lineages into CD4^+^T cells, CD8^+^T cells, Cycling T cells, and γδ T cells. **(B)** Dot plot showing the expression profiles of canonical marker genes across defined T cell subsets (Tn, Tcm, Trm, Tex, MAIT, IEL, Tem, and Temra). **(C)** Pearson correlation analysis between *CX3CR1* and *CEBPB* expression in the TCGA-COAD cohort (n = 512). No significant correlation was observed (*R* = 0.07, *P* = 0.10). **(D)** Differential heatmaps illustrating the shift in interaction frequency (left) and signaling intensity (right) across cell populations. Red shading signifies augmented crosstalk in the post-treatment cohort relative to baseline. Notably, the highlighted rows and columns delineate robustly enhanced communication between Temra cells and myeloid compartments including macrophages and dendritic cells. **(E)** Bar plots quantifying the aggregate number and cumulative strength of inferred intercellular interactions comparing pre-treatment and post-treatment samples. The substantial elevation in both metrics underscores the global intensification of immune crosstalk within the tumor microenvironment following PD-1 blockade. **(F)** Relative information flow analysis identifying signaling pathways preferentially enriched in pre-treatment (red) versus post-treatment (blue) tissues. The transition from MIF-dominant signaling at baseline toward adhesion-related pathways such as CD99 and ADGRE5 following therapy underscores a fundamental remodeling of the tumor immune microenvironment.

Supplementary Figure 3 Gating strategy for the identification and functional analysis of CX3CR1^+^CD8^+^T cells by flow cytometry. **(A)** Representative plots illustrating the gating hierarchy to identify live, single CD45^+^CD3^+^CD8^+^T cells. The CX3CR1^+^ subset was further analyzed for intracellular expression of GZMB, IFN-γ and TNF-α. **(B, C)** Longitudinal quantification of the frequency and functional status (GZMB, IFN-γ, TNF-α) of CX3CR1^+^CD8^+^T cells in the spleen **(B)** and lymph nodes **(C)**. Data represent mean ± SEM (n = 7). **P* < 0.05, ***P* < 0.01, ****P* < 0.001 (Two-way ANOVA).

Supplementary Figure 4 Transcriptional characterization of CD8^+^T cell subsets and molecular remodeling induced by PD-1 blockade. **(A)** Dot plot displaying the expression of canonical marker genes across defined CD8^+^T cell clusters. Selected markers for stemness (*Tcf7*, *Sell*), exhaustion (*Pdcd1*, *Havcr2*), and cytotoxicity (*Gzmb*) are indicated to verify cluster identities. **(B)** Mirror volcano plot depicting DEGs between anti-PD-1 treated and control groups. Red dots indicate upregulated genes (e.g., *Cxcl9*, *Ccl12*, *Cd36*), while blue dots indicate downregulated genes (e.g., *Gzmk*, *Fasl*). **(C)** Heatmaps illustrating the differential number (left) and cumulative intensity (right) of inferred cell-cell communications. The red color scale signifies elevated interaction levels following PD-1 blockade. Specifically, the intersection of CD8^+^Temra (sender population) and myeloid compartments including Macrophages and DCs (receiver populations) identifies the most significant amplification in interaction strength, providing evidence for a robustly coordinated immune response post-treatment. **(D)** Bar charts summarizing the aggregate count and integrated intensity of signaling pathways. The therapy-induced increase in both metrics underscores the intensified crosstalk within the tumor microenvironment of the αPD-1 group, suggesting a more robustly coordinated multicellular response. **(E)** Relative information flow analysis identifying distinct signaling pathways preferentially enriched in the control (red) and αPD-1 (teal) cohorts. These data demonstrate a therapy-induced shift in the tumor microenvironment, where baseline-dominant immunosuppressive pathways such as *TGFβ* and *MIF* are superseded by pro-inflammatory and recruitment-associated cues including *CXCL*, *CCL*, *IFN-II*, and *CD86* signaling following PD-1 blockade.
