# Supplementary figures and images for: CX3CR1 identifies a potent effector CD8+ T cell subset associated with anti-PD-1 therapeutic efficacy in colorectal cancer
Source: Front Immunol. 2026 Mar 18;17:1770119. doi: 10.3389/fimmu.2026.1770119 (PMC13039036; doi:10.3389/fimmu.2026.1770119)

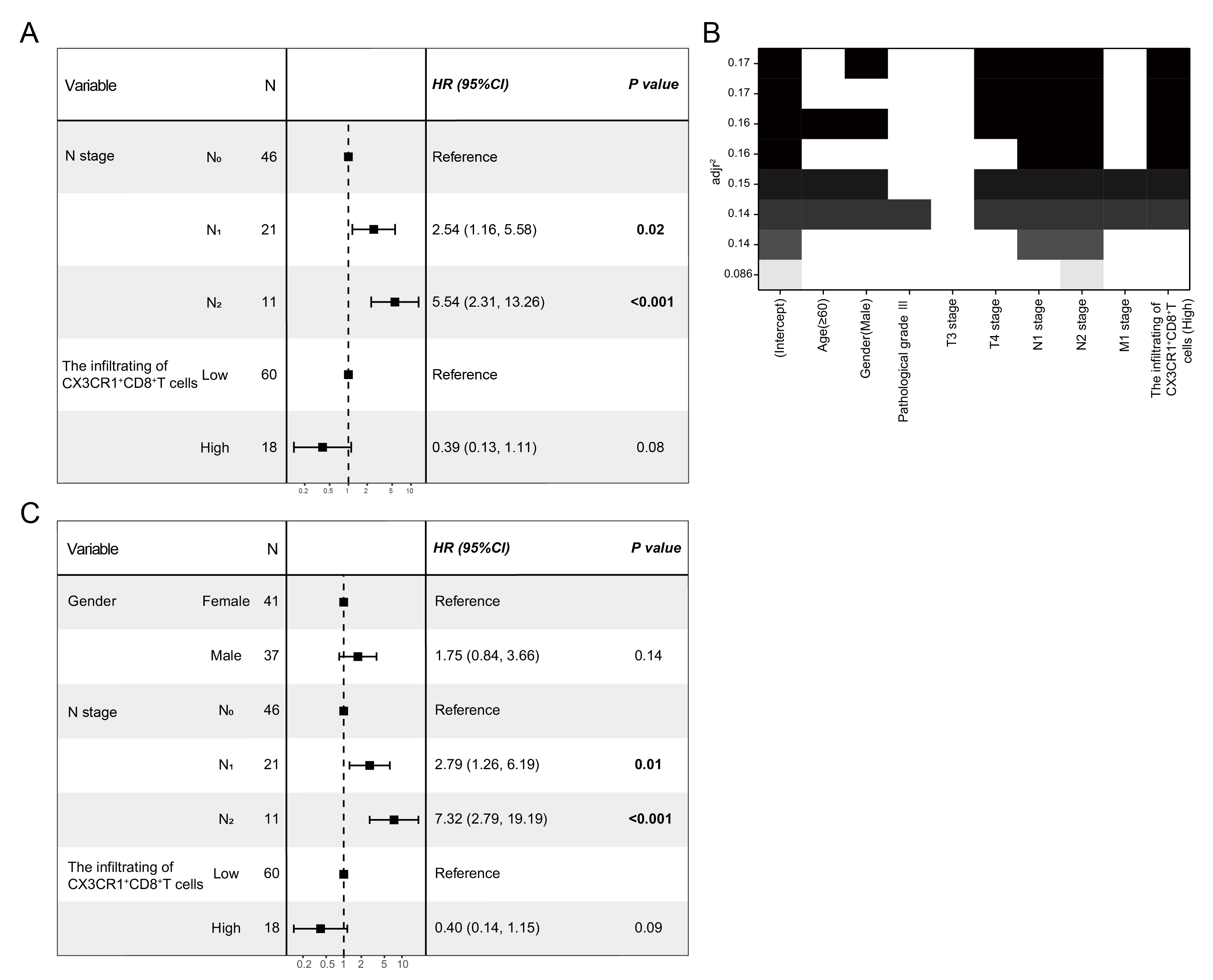

Supplement: Supplementary Figure 1 — Identification of independent prognostic factors for CRC patients via multi-dimensional statistical approaches. (A) Forest plot of the multivariate Cox regression model incorporating significant univariate predictors. (B) Variable selection using BSR based on the adjusted R2 criterion. The optimal model with the highest adjusted R2 is identified. (C) Multivariate Cox regression model constructed using BSR-selected variables (Gender, N stage, and CX3CR1+CD8+T cell infiltration), confirming the protective value of the immune subset (HR = 0.40). [file Image1.jpeg]

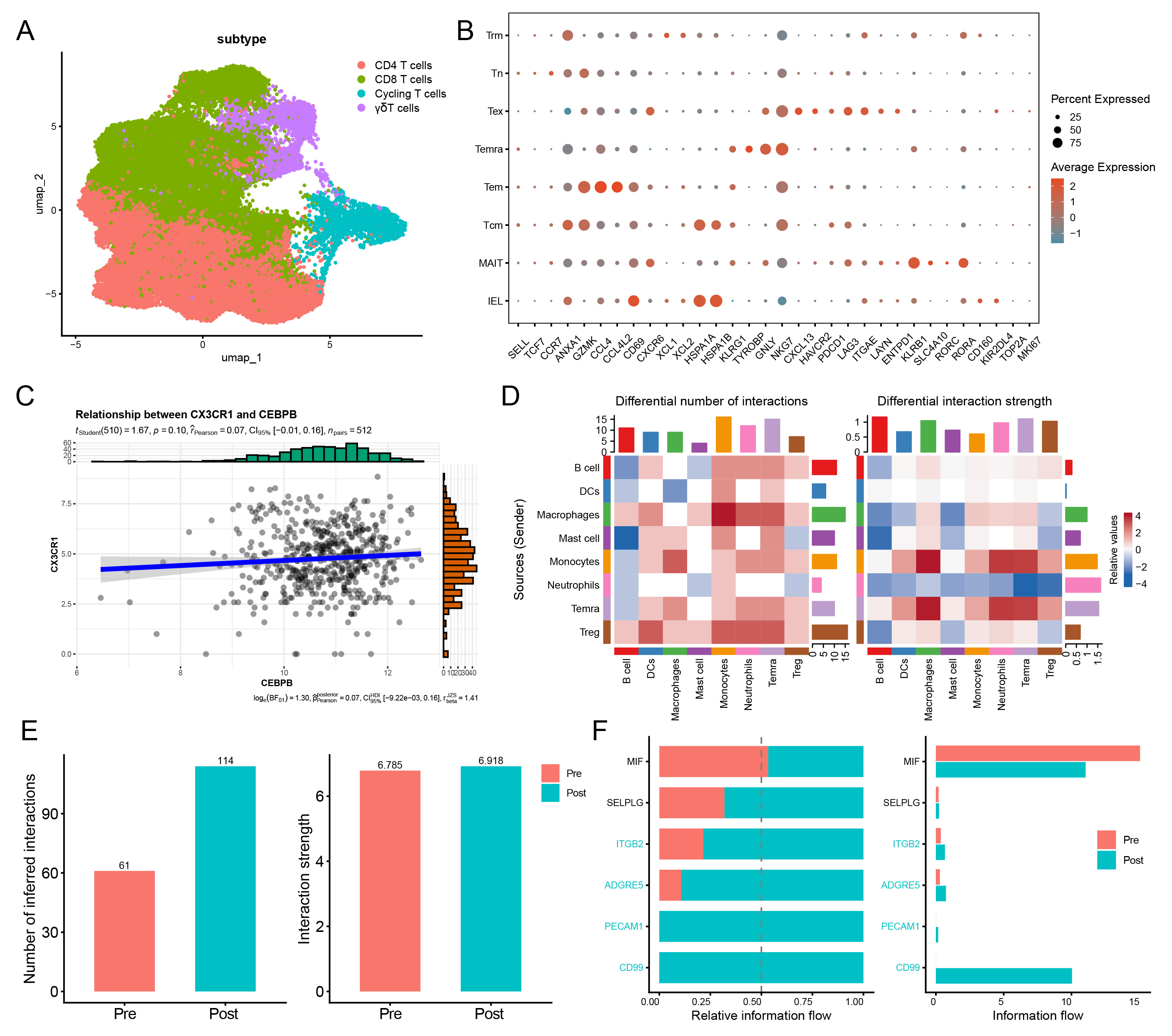

Supplement: Supplementary Figure 2 — Characterization of T cell subtypes and validation of CEBPB correlation in CRC. (A) UMAP visualization of the total T cell compartment isolated from tumor tissues, resolving major lineages into CD4+T cells, CD8+T cells, Cycling T cells, and γδ T cells. (B) Dot plot showing the expression profiles of canonical marker genes across defined T cell subsets (Tn, Tcm, Trm, Tex, MAIT, IEL, Tem, and Temra). (C) Pearson correlation analysis between CX3CR1 and CEBPB expression in the TCGA-COAD cohort (n = 512). No significant correlation was observed (R = 0.07, P = 0.10). (D) Differential heatmaps illustrating the shift in interaction frequency (left) and signaling intensity (right) across cell populations. Red shading signifies augmented crosstalk in the post-treatment cohort relative to baseline. Notably, the highlighted rows and columns delineate robustly enhanced communication between Temra cells and myeloid compartments including macrophages and dendritic cells. (E) Bar plots quantifying the aggregate number and cumulative strength of inferred intercellular interactions comparing pre-treatment and post-treatment samples. The substantial elevation in both metrics underscores the global intensification of immune crosstalk within the tumor microenvironment following PD-1 blockade. (F) Relative information flow analysis identifying signaling pathways preferentially enriched in pre-treatment (red) versus post-treatment (blue) tissues. The transition from MIF-dominant signaling at baseline toward adhesion-related pathways such as CD99 and ADGRE5 following therapy underscores a fundamental remodeling of the tumor immune microenvironment. [file Image2.jpeg]

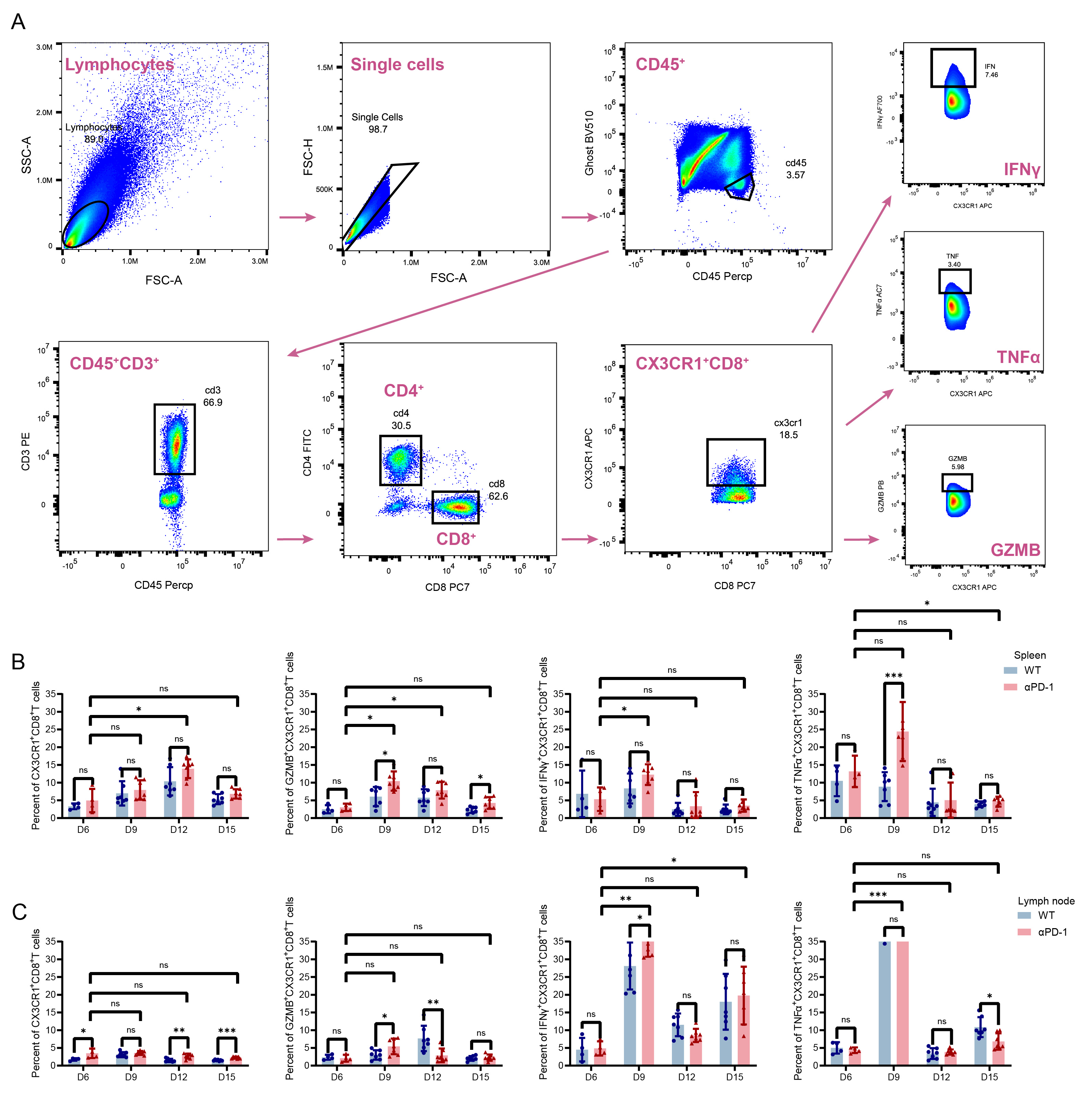

Supplement: Supplementary Figure 3 — Gating strategy for the identification and functional analysis of CX3CR1+CD8+T cells by flow cytometry. (A) Representative plots illustrating the gating hierarchy to identify live, single CD45+CD3+CD8+T cells. The CX3CR1+ subset was further analyzed for intracellular expression of GZMB, IFN-γ and TNF-α. (B, C) Longitudinal quantification of the frequency and functional status (GZMB, IFN-γ, TNF-α) of CX3CR1+CD8+T cells in the spleen (B) and lymph nodes (C). Data represent mean ± SEM (n = 7). *P < 0.05, **P < 0.01, ***P < 0.001 (Two-way ANOVA). [file Image3.jpeg]

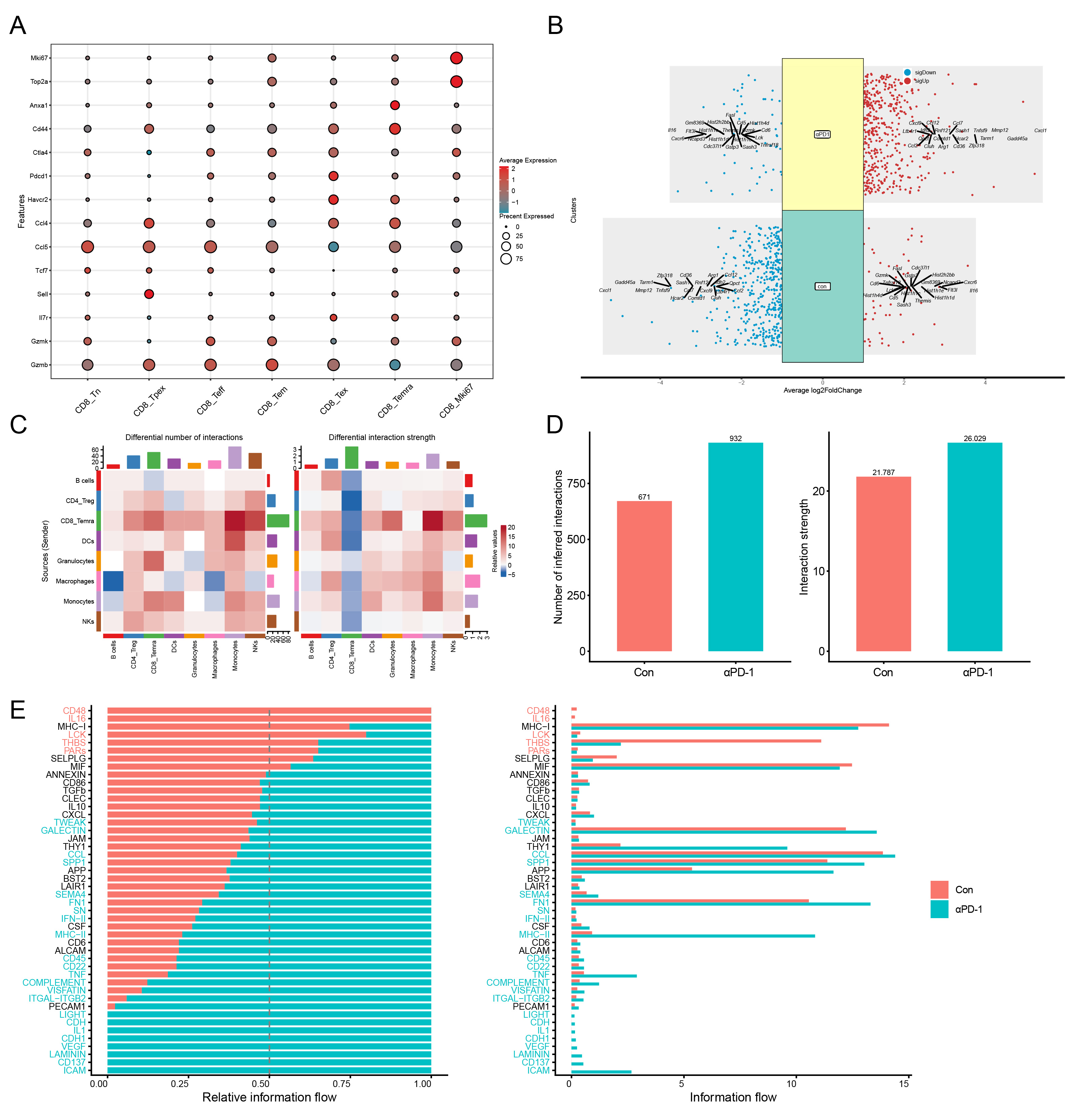

Supplement: Supplementary Figure 4 — Transcriptional characterization of CD8+T cell subsets and molecular remodeling induced by PD-1 blockade. (A) Dot plot displaying the expression of canonical marker genes across defined CD8+T cell clusters. Selected markers for stemness (Tcf7, Sell), exhaustion (Pdcd1, Havcr2), and cytotoxicity (Gzmb) are indicated to verify cluster identities. (B) Mirror volcano plot depicting DEGs between anti-PD-1 treated and control groups. Red dots indicate upregulated genes (e.g., Cxcl9, Ccl12, Cd36), while blue dots indicate downregulated genes (e.g., Gzmk, Fasl). (C) Heatmaps illustrating the differential number (left) and cumulative intensity (right) of inferred cell-cell communications. The red color scale signifies elevated interaction levels following PD-1 blockade. Specifically, the intersection of CD8+Temra (sender population) and myeloid compartments including Macrophages and DCs (receiver populations) identifies the most significant amplification in interaction strength, providing evidence for a robustly coordinated immune response post-treatment. (D) Bar charts summarizing the aggregate count and integrated intensity of signaling pathways. The therapy-induced increase in both metrics underscores the intensified crosstalk within the tumor microenvironment of the αPD-1 group, suggesting a more robustly coordinated multicellular response. (E) Relative information flow analysis identifying distinct signaling pathways preferentially enriched in the control (red) and αPD-1 (teal) cohorts. These data demonstrate a therapy-induced shift in the tumor microenvironment, where baseline-dominant immunosuppressive pathways such as TGFβ and MIF are superseded by pro-inflammatory and recruitment-associated cues including CXCL, CCL, IFN-II, and CD86 signaling following PD-1 blockade. [file Image4.jpeg]
